# Immunological Biomarkers as Predictors of Treatment Response in Psychotic Disorders

**DOI:** 10.3390/jpm13091382

**Published:** 2023-09-15

**Authors:** Elif Bayram Orbe, Michael Eriksen Benros

**Affiliations:** 1 Copenhagen Research Centre for Biological and Precision Psychiatry, Mental Health Centre Copenhagen, Copenhagen University Hospital, 2900 Hellerup, Denmark; 2 Department of Clinical Medicine, Faculty of Health and Medical Sciences, University of Copenhagen, 1172 Copenhagen, Denmark

**Keywords:** schizophrenia, psychosis, immunology, anti-inflammatory drugs, personalized medicine

## Abstract

Psychotic disorders, notably schizophrenia, impose a detrimental burden on both an individual and a societal level. The mechanisms leading to psychotic disorders are multifaceted, with genetics and environmental factors playing major roles. Increasing evidence additionally implicates neuro-inflammatory processes within at least a subgroup of patients with psychosis. While numerous studies have investigated anti-inflammatory add-on treatments to current antipsychotics, the exploration of immunological biomarkers as a predictor of treatment response remains limited. This review outlines the current evidence from trials exploring the potential of baseline inflammatory biomarkers as predictors of the treatment effect of anti-inflammatory drugs as add-ons to antipsychotics and of antipsychotics alone. Several of the studies have found correlations between baseline immunological biomarkers and treatment response; however, only a few studies incorporated baseline biomarkers as a primary endpoint, and the findings thus need to be interpreted with caution. Our review emphasizes the need for additional research on the potential of repurposing anti-inflammatory drugs while utilizing baseline inflammatory biomarkers as a predictor of treatment response and to identify subgroups of individuals with psychotic disorders where add-on treatment with immunomodulating agents would be warranted. Future studies investigating the correlation between baseline inflammatory markers and treatment responses can pave the way for personalized medicine approaches in psychiatry centred around biomarkers such as specific baseline inflammatory biomarkers in psychotic disorders.

## 1. Introduction

Psychotic disorders vary in terms of symptoms, severity, and prognosis. The most common psychotic disorder is schizophrenia with a lifetime prevalence of 0.75% [1]. To treat and prevent psychotic episodes, patients often take antipsychotic drugs for years, which can have multiple side effects. Moreover, a considerable percentage of the patients have insufficient treatment response with current treatments. According to a recent meta-analysis on first-episode psychosis (FEP) and first-episode schizophrenia (FES), the rate of treatment-resistant schizophrenia (TRS) was 17.8% in the FEP group and 24.4% in the FES group, while the overall rate of treatment resistance was 22.8% [2]. There are two leading theories of TRS: one is normal dopamine function, but with the dysregulation of the serotonin, glutamate or inflammatory pathways, while the second theory proposes that dopaminergic supersensitivity leads to TRS over time [3]. This calls for new treatment options and advocates for more personalized treatments preferentially based on the early identification of objective markers, such as immunological biomarkers [4,5,6]. 

The aetiology behind psychotic disorders including schizophrenia is not yet fully understood [7]. It is known that there is a substantial genetic component [8] and that environmental factors contribute to the development of psychotic disorders [9]. Moreover, the past decades have given rise to mounting evidence supporting the role of immunological processes in the development of psychotic disorders [10,11,12,13]. This includes elevated levels of a broad range of immunological biomarkers such as cytokines and white blood cell count (WBC) in both blood and cerebrospinal fluid (CSF), indicating a central role of the immune system in developing or maintaining psychosis in at least a subgroup of patients [14,15,16,17,18,19]. Moreover, studies have investigated the increased density and activation of microglia in the brain, indicative of neuroinflammation among subgroups [20], although a recent meta-analysis found no significant association with the volume of the distribution of translocator protein (TSPO), which is believed to reflect microglial activation, though recent research has indicated that it might more reflect the density of inflammatory cells [21,22]. Some studies suggest that neuroinflammation may only be present during the first episode of psychosis, meaning that the effect of anti-inflammatory add-on treatment might not be as efficient in non-acute patients [12,23,24,25], while other studies hypothesize that patients with schizophrenia and chronic low-grade peripheral inflammation will benefit the most [26]. Several studies over the last years have reported signs of peripheral inflammation associated with increased levels of negative symptoms of schizophrenia and increased severity of cognitive deficits [27]. Almost one-third of patients with schizophrenia suffer deficit schizophrenia [28], i.e., patients with primary and persistent negative symptoms, and studies have found pro-inflammatory markers associated with this syndrome [29]. Moreover, meta-analyses of cytokines in both blood and cerebrospinal fluid have shown mental state-dependent associations with more pronounced alterations of cytokines and lymphocytes during acute episodes, e.g., the first episode of psychosis or during relapse, as compared to post-treatment periods [14,16,18,30,31]. Meta-analyses comparing patients with psychotic disorders with healthy controls have shown increased levels of lymphocytes [30], monocytes [32], an increased neutrophil/lymphocyte ratio (NLR) [33,34], and higher concentrations of C-reactive protein (CRP) [35] among the patient group. However, previous register-based studies investigating first-time diagnoses of schizophrenia and at-admission baseline CRP and WBC found no association with the risk of subsequent psychiatric admissions [36] or treatment resistance among patients with increased baseline CRP (>3 mg /L) [37]. A recent meta-analysis showed that drugs possessing either primary or pleiotropic anti-inflammatory properties, as an add-on to the antipsychotic treatment of psychotic disorders, improve the negative and positive symptoms and slow cognitive decline compared to placebos [38]. In addition, several previous meta-analyses on add-on drugs with either primary or pleiotropic anti-inflammatory properties have been performed, but none of the previous meta-analyses investigated baseline immune biomarkers and treatment effects [39,40,41,42,43,44,45,46,47,48,49,50,51,52,53,54,55,56].

In studies of antipsychotics, a meta-analysis has shown changes in cytokine levels compared to baseline in first-episode psychotic patients and post-treatment with antipsychotics, where decreases in pro-inflammatory interleukin-1 beta (IL-1β), interleukin-6 (IL-6), interferon-gamma (IFN-γ), tumor necrosis factor alpha (TNF-α), and anti-inflammatory interleukin-4 (IL-4) and interleukin-10 (IL-10) were observed, while there was no difference in interleukin-2 (IL-2) and interleukin-17 (IL-17) [57]. In addition, a meta-analysis has investigated differences in IL-6 post-clozapine treatment vs. other antipsychotics, showing increased levels of IL-6 in the clozapine-treated group, again suggesting different effects on inflammatory cytokines among individual antipsychotic drugs [58].

## 2. Method

In this narrative review, we will present an overview of the conducted trials on anti-inflammatory add-on treatment to antipsychotics in psychotic disorders, where immunological biomarkers were analysed to assess their ability to predict treatment effects. Additionally, we will present an overview of trials investigating baseline immunological markers as predictors of response to antipsychotic treatment to establish an overview of relevant biomarkers in the prediction of treatment response to antipsychotics. We only included trials in which psychotic disorders were diagnosed according to a diagnostic classification from either the Diagnostic Statistical Manual of Mental Disorders (DSM) or the International Classification of Diseases (ICD). The databases searched for this review were PubMed, CENTRAL, ClinicalTrials.gov, PsycINFO and EMBASE. 

The purpose of this review is to identify promising candidate biomarkers for the identification of those patients who would likely benefit from treatment with anti-inflammatory add-on treatment to antipsychotics, thus coming one step closer to achieving precision psychiatry and new, innovative treatments.

## 3. Results

Twenty-three studies were identified: nine studies on anti-inflammatory add-on drugs to antipsychotics and thirteen studies on antipsychotics. All studies on anti-inflammatory add-on drugs are randomized controlled trials (RCTs), whereas the majority of the studies on antipsychotic drugs are observational. We will summarize these findings under two headings: studies of immunological biomarkers as predictors of treatment response to anti-inflammatory add-on treatment to antipsychotics and studies of baseline immunological biomarkers and correlations with treatment response to antipsychotics.

### 3.1. Studies of Immunological Biomarkers as Predictors of Treatment Response to Anti-Inflammatory Add-on Treatment to Antipsychotics (Table 1)

#### 3.1.1. Aspirin

Aspirin is an irreversible inhibitor of the enzymes cyclooxygenase (COX) 1 and—though with lower affinity—COX 2, thereby reducing the production of prostaglandins, IL-1β, TNF-α, and IL-6. It inhibits the mitogen-activated protein kinase (MAPK) and nuclear factor kappa-light chain enhancer of activated B-cells (NF-κB) pathways and upregulates the differentiation of regulatory T-cells [59]. Laan et al., 2010 [60], studied Aspirin in 70 patients (58 analysed) with schizophrenia, schizoaffective disorder, or schizophreniform disorder with a minimum score of 60 on the positive and negative syndrome scale (PANSS), where higher scores indicate more severe symptoms; they investigated T helper cells (T_H_) at baseline in conjunction with levels of specific cytokines corresponding to the levels of T_H_1 and T_H_2. They investigated the ratio of proinflammatory T_H_1 response, represented by levels of IFN-γ, over anti-inflammatory T_H_2 response, represented by levels of IL-4, thus the described T_H_1/T_H_2 cytokine ratio in the paper is indicated by the median IFN-γ/IL-4-ratio [61]. Aspirin was expected to increase the T_H_1/T_H_2 ratio due to the downregulation of anti-inflammatory T_H_2 cells and the produced cytokines in favour of proinflammatory T_H_1. Analyses of the scores were repeated in subgroups of patients defined by the median T_H_1/T_H_2 ratio, which showed that patients in the lowest T_H_1/T_H_2 group had higher (*p* = 0.018) treatment efficacy with a mean difference of total PANSS of 7.47 compared to the observed mean difference of total PANSS of 2.39 in the group with the highest T_H_1/T_H_2. The authors also separately measured IL-4, IL-10, and IFN- γ, which did not appear to modify treatment efficacy. 

Weiser et al. 2021 [48] presented data from two separate studies referred to as study 1 (inclusions in 2011) and study 2 (inclusions in 2014–2016) on the use of aspirin in patients with schizophrenia or schizoaffective disorder who had at least two prior psychotic episodes and/or had been continually ill for at least 6 months. Study 1 (*n* = 200) was conducted as a part of a larger four-arm clinical trial with pramipexole (*n* = 100), minocycline (*n* = 100), aspirin (*n* = 100), and a placebo arm (*n* = 100). In study 1, early post hoc subgroup analyses suggested that aspirin improved PANSS-positive scores by an effect size of 0.6 among the patients in the highest tertile of baseline CRP; however, the authors did not specify how many patients were included in this subgroup analysis. When the authors conducted a formal heterogeneity test for effect modification by baseline CRP on the full sample of 200, the result was not significant. To investigate the utility of CRP as a biomarker of the prediction of treatment effect with aspirin, the authors conducted a new study in a new group of patients with baseline CRP > 1 mg/L and, in addition to the same diagnostic criteria as study 1, patients in study 2 had to have a score of ≥4 (moderate or worse) on two or more of the PANSS items of delusions, hallucinatory behaviours, conceptual disorganization, and suspiciousness/persecution (study 2 *n* =160). In study 2, the authors did meet the power calculation (it was estimated that at least 120 patients with baseline CRP > 1 mg/L were needed to obtain 90% power). The results based on baseline CRP levels above 1 mg/L were not significant compared to placebo in study 2. They also analysed between-group differences (aspirin vs. placebo) in PANSS total at the end of treatment according to the strata of baseline CRP above and below 4 mg/L in both studies 1 and 2, which remained not significant.

#### 3.1.2. Celecoxib 

Celecoxib is a selective COX-2 inhibitor—about 10–20 times more selective for COX-2 than for COX-1 [62]. In a study with 50 patients with schizophrenia, Müller et al., 2004 [63], investigated add-on celecoxib and found an overall significant effect on the mean improvement in total PANSS. In a secondary analysis, patients were divided into responders and non-responders according to a criterion of at least 30% improvement of the PANSS total scale. The study found a significant difference in lower soluble tumor necrosis factor receptor 1 (sTNF-R1) levels at baseline and response to treatment—meaning that the responders had significantly lower sTNF-R1 compared to non-responders. However, none of the other immune markers investigated at baseline (cluster of differentiation (CD) 4+, CD8+, CD19+, soluble interleukin-2 receptor (IL-2R)) showed a statistically significant relationship with treatment response to celecoxib. 

#### 3.1.3. Minocycline 

Deakin et al., 2018 [64], studied adjunctive minocycline (*n* = 129 analysed) in patients with first-episode schizophrenia and schizoaffective disorder diagnosed within 5 years. Minocycline is a semisynthetic tetracycline analogue capable of crossing the blood–brain barrier due to its small size and higher lipid solubility [65]. Minocycline has been shown to reduce levels of IL-1β and TNF-α [66], inhibit the COX 2 and MAPK and NF-κB pathways [67], and impair protein kinase C, thus suppressing T-cell proliferation and activation and reducing T-cell-microglia interaction [67,68]. Deakin et al. [64] found no effect on treatment outcomes in the 129 patients when stratifying the groups based on above- or below-median hs-CRP or IL-6 concentrations at baseline. 

#### 3.1.4. Tocilizumab 

Tocilizumab is a monoclonal antibody that competitively inhibits the binding of IL-6 to its receptor IL-6R, which inhibits the entire receptor complex and its pro-inflammatory properties, thus leading to increasing regulatory T-cell differentiation and reduced levels of CRP [69,70]. A study by Girgis et al., 2018 [71], on tocilizumab with 36 patients with schizophrenia or schizoaffective disease with a PANSS total of >60 found, before adjusting for multiple testing, that higher GM-CSF at baseline correlated with better treatment efficacy in PANSS total and PANSS positive. However, after adjusting for multiple testing, the associations were no longer significant between treatment effect and baseline CRP, cytokine levels (IL-1β, IL-6, interleukin-8 (IL-8), IL-10, interleukin-12 (IL-12), interleukin-17a (IL 17a), TNF-α and IFN-γ), or granulocyte-macrophage colony-stimulating factor (GM-CSF). 

#### 3.1.5. N-acetylcysteine (NAC)

N-acetylcysteine is a cysteine prodrug and glutathione (GSH) precursor that has been shown to reduce levels of IL-1β, IL-8, TNF-α, and IL-6 as well as inhibit NF-κB pathways [72]. Conus et al., 2018 [73], studied N-acetylcysteine add-on treatment to antipsychotics with 61 patients with psychosis within the past 12 months; they investigated whether GSH/redox markers could be a valid biomarker for the prediction of treatment response. The study found that in patients with baseline GSH peroxidase activity higher than 22.3 U/g (36% of patients included in the study), PANSS positive scores improved.

#### 3.1.6. Pravastatin

Vincenzi et al., 2014 [74] (*n* = 49 analysed), investigated pravastatin, a drug that competitively inhibits HMG-COA reductase [75] and has been shown to reduce levels of IL-1β, TNF-α, and prostaglandin E2 [76,77] and downregulates the MAPK and NF-κB pathways [78]; they did not find any significant difference on psychopathological or cognitive measurements between the intervention group and placebo group, but when in secondary analyses of a subgroup of participants with CRP above 2 mg /L, pravastatin was associated with an improvement from baseline on cognition in up to 6 weeks. Over time, the results did not remain significant. No significant change in psychopathology was found [74].

#### 3.1.7. Prednisolone

Prednisolone is a potent glucocorticosteroid that induces leucocytosis, the elevation of neutrophils, and reduces levels of TNF-α, IL-6, IL-8, monocyte chemoattractant protein 1 (MCP-1), and CRP (the decrease in CRP levels are thought to be mediated by the inhibition of IL-6, which is a strong CRP stimulator) while increasing levels of IL-10 [79]. Nasib et al., 2021 [80] (*n* = 39 patients analysed), investigated prednisolone, where levels of high sensitivity CRP (hs-CRP) were available in 33 patients with a higher baseline PANSS total and with elevated CRP > 3.9 mg/L (*n* = 20, 12 in the intervention group and 8 in the placebo group), but there was no significant effect on the end-of-treatment PANSS total, nor when subdividing into PANSS positive, PANSS negative, or PANSS general. 

**Table 1:** Overview of the 9 studies with anti-inflammatory add-on drugs included in this review.

Author	Country	Disease	Treatment	Treatment Length	N Treatment Analysed	N Control Analysed	Baseline Marker	Conclusion
Laan 2010 [60]	The Netherlands	SCZ < 10 years SCFD < 10 years SCAD < 10 years	Aspirin 1 g/day	3 months	27	31	T_H_1/T_H_2 cytokine ratio IL-4, IL-10, and IFN-γ	The lower T_H_1/T_H_2 ratio group had higher efficacy with a mean difference of total PANSS of 7.47 compared to 2.39 in the highest ↑T_H_1/T_H_2. IL-4, IL-10, and IFN- γ did not substantially modify treatment efficacy.
Weiser 2021 [48] Study 1	Study 1: 18 sites in Romania and 1 site in Moldova	Study 1: SCZ, SCAD, and >2 psychotic episodes or ill > 6 month	Aspirin	16 weeks	100	100	CRP > 1 mg/L	No significant difference was found.
Weiser 2021 [48] Study 2	Study 2: 30 sites in Romania	Study 2: same as study 1 + baseline CRP > 1 mg/L	Aspirin	16 weeks	80	80	CRP > 1 mg/L	No significant difference was found.
Müller 2004 [63]	Germany	SCZ	Celecoxib 400 mg/day	5 weeks	25	25	sTNF-R1, CD4+, CD8+, CD19+, sIL-2R	Responders had significantly lower sTNF-R1 compared to non-responders. CD4+, CD8+, CD19+, sIL-2R did not affect treatment response.
Deakin 2018 [64]	UK	FEP SCZ, SCFD, SCAD, or psychosis < 5 years	Minocycline 200 mg/day week 1–2, then 300 mg/day	1 year	64	65	Above or below median hs-CRP or IL-6 at baseline	No effect on treatment outcome.
Girgis 2018 [71]	USA	SCZ or SCAD	Tocilizumab 8 mg/kg per month	3 months	19	17	CRP, IL-1β, IL-6, IL-8, IL-10, IL-12, IL 17a, TNF-α, and IFN-γ at baseline	No effect on treatment response.
Conus 2018 [73]	USA and Switzerland	Psychosis < 1 year	N-acetylcysteine 2.7 g/day	6 months	31	30	GSH peroxidase activity > 22.3 U/g	Baseline GSH peroxidase activity higher than 22.3 U/g was associated with improvement in PANNS positive score.
Vincenzi 2014 [74]	USA	SCZ or SCAD	Pravastatin 40 mg/day	12 weeks	24	25	CRP > 2 mg/L	No significant effect on outcome.
Nasib 2021 [80]	Netherlands and Belgium	SCZ, SCFD, SCAD, or psychosis NOS < 7 years	Prednisolone 1 Week: 40 mg/day for 3 days, then 30 mg/day for 4 days. From week 2 until week 6 diminish by 5 mg prednisolone each week.	6 weeks	20	19	hs-CRP	No significant effect on the outcome.

SCZ = schizophrenia; SCFD = schizophreniform disorder; SCAD = schizoaffective disorder; psychosis NOS = psychosis not otherwise specified; FEP = first-episode psychosis; FES = first-episode schizophrenia.

### 3.2. Studies of Baseline Immunological Biomarkers and Correlations with Treatment Response to Antipsychotics (Table 2)

#### 3.2.1. Studies on Specific Antipsychotic Drugs

##### Olanzapine

Ma et al., 2021 [81], studied 82 male patients with first-episode schizophrenia (duration < 5 years) in a case study undergoing 4 weeks of olanzapine treatment. They classified patients into effective (*n* = 40), i.e., responders, and ineffective groups (*n* = 42), i.e., non-responders, based on the PANSS reduction rate. They found that responders had lower baseline IL-1β (SE = −0.08, OR = 0.93 [0.86–1.00]; *p* = 0.04) and lower baseline IL-17 (SE = −0.05, OR = 0.95 [0.90–0.99]; *p* = 0.03), while no correlation was found for IL-6, hs-CRP, or transforming growth factor beta-1 (TGF-β1). 

Another observational study by Hatziagelaki et al., 2019 [82], on 14 drug-naïve patients with first-episode schizophrenia before and after the initiation of 8 weeks of olanzapine monotherapy treatment, found that the negative symptom items on the PANSS correlated positively with baseline IL-6 and interleukin-27 (IL-27) levels, while this association was not significant for brain-derived neurotrophic factor (BDNF), interleukin-2 (IL-2), interleukin-17F (IL-17F), interleukin-17A (IL-17A), interleukin-22 (IL-22), IL-1β, interleukin-21 (IL-21), interleukin-23 (IL-23), IL-4, IFN-γ, TGF-β1, transforming growth factor beta-2 (TGF-β2), and transforming growth factor beta-3 (TGF-β3). 

In a third observational study, Lin et al., 2017 [83], investigated baseline MCP-1, IL-1β, and TNF-α in 38 patients with schizophrenia receiving olanzapine monotherapy and found no significant correlation with subsequent treatment effects. This investigation was part of a combined study of 64 patients with schizophrenia, where the remaining 26 received risperidone. 

##### Risperidone

Lin et al., 2017 [83], investigated 26 patients with schizophrenia receiving 4 weeks of treatment with risperidone monotherapy in an observational study. They found a negative correlation between pre-treatment MCP-1 levels (r = −0.658; *p* = 0.0003) and general PANSS (PANNS-G) score reduction, while the correlation was not significant for total PANSS. IL-1β and TNF-α were not significantly correlated with treatment effect. This investigation was part of a combined study of 64 patients with schizophrenia, where the remaining 38 received olanzapine.

##### Amisulpride

As part of the OPTiMiSE study investigating the first episode of schizophrenia, schizophreniform disorder, or schizoaffective disorder undergoing 4 weeks of amisulpride treatment to investigate symptomatic remission, Susai et al., 2023 [84], investigated baseline complement proteins using proteomics and found that baseline levels of 11 complement proteins (complement C1R subcomponent (C1R), complement C1s subcomponent (C1S), complement C2 (C2), complement C5 (C5), complement component C6 (C6), complement component C7 (C7), complement component C8 alpha chain (C8A), complement component C8 beta chain (C8B), complement factor I (CFI), ficolin-3 (FCN3), and mannose-associated serine protease 1 (MASP-1)) were significantly associated with a reduction in total PANSS score, while a substantial group of complement proteins they investigated was not associated with outcome. 

The study by Martinuzzi et al., 2019 [85], was likewise part of the OPTiMiSE study, where first-episode psychosis patients underwent 4 weeks of amisulpride treatment. The study investigated the following immunological biomarkers: IL-1α, IL-1β, IL-2, IL-4, IL-5, IL-6, IL-7, IL-8, IL-10, IL-12p40, IL- 12p70, IL-13, IL-15, IL-16 IL- 17, IL-18, IL-21, IL-23, IL-27, IFN-γ, chemokines (C-C motif chemokine ligand (CCL)-2, CCL3, CCL4, CCL11, CCL13, CCL17, CCL19, CCL20, CCL22, CCL26, CCL27, and C-X3-C motif chemokine ligand (CX3CL)-1, CXCL10, CXCL11, CXCL12), TNF-α, TNF-β, GM-CSF, vascular endothelial growth factor (VEGF), CRP, serum amyloid A protein (SAA), soluble intercellular adhesion molecule 1 (sICAM-1), and soluble vascular adhesion molecule 1 (sVCAM-1). Initially, their study found no correlation with biomarkers at baseline or response to treatment. After clustering patients within four groups based on symptoms, they found that patients in cluster C1A had a higher risk of non-remission correlating to lower serum interleukin-15 (IL-15) at baseline and higher serum C-X-C motif chemokine 12 (CXCL12) at baseline. The cluster of patients was based on PANNS symptomatology and the C1A group had more severe negative and general psychopathology as well as more prominent positive psychopathology.

#### 3.2.2. Studies on Various Antipsychotics, Treating Them as a Unified Group for Analysis

Although several other studies investigating the correlation between baseline inflammatory biomarkers and treatment effects were identified, the specification of effect by each antipsychotic drug individually is lacking.

##### Risperidone, Olanzapine, and Haloperidol

Crespo-Facorro et al., 2008 [86], investigated 56 drug-naïve first-episode psychosis patients randomly assigned to 6 weeks of risperidone (*n* = 16), olanzapine (*n* = 20), or haloperidol (*n* = 20) treatment and the effect on interleukin-12 (IL-12) production. No significant associations between IL-12 production (measured by IL-12p70) and the severity of clinical symptoms at baseline, at 6 weeks, or with changes in psychopathology over the 6 weeks were found.

##### Risperidone, Olanzapine and Quetiapine

Pae et al., 2006 [87], conducted an observational study with 8 weeks of risperidone (*n* = 14), olanzapine (*n* = 19), or quetiapine (*n* = 2) in drug-naïve (>2 months) schizophrenia patients (*n* = 35). They analysed patients in subgroups of responders (20% or more decrease in total PANSS from baseline) or non-responders, and they found no significant differences for baseline IL-2, interleukin-13 (IL-13), IL-6, IL-10, and TNF-α. Additionally, they found that baseline IL-6 was correlated with a change in the PANSS-G score (r = 0.449, *p* = 0.044). No other correlation was found between changes in the PANSS and baseline cytokine levels. 

##### Risperidone, Olanzapine, Quetiapine, Aripiprazole, Ziprasidone, Perphenazine, and Haloperidol

Zhang et al., 2023 [88], randomly assigned (1:1:1:1:1:½:½) patients with schizophrenia (*n* = 2598) to risperidone, olanzapine, quetiapine, aripiprazole, ziprasidone, perphenazine, or haloperidol. In the study, patients with a PANSS reduction greater than 25% were defined as responders and the rest were non-responders. They compared the difference in total WBC between responders and non-responders and found that responders, based on different PANSS scores, all had lower total WBCs at baseline (*p* < 0.05).

##### Risperidone and Haloperidol

Zhang et al., 2004 and 2005 [89,90], randomly assigned 78 patients with schizophrenia to risperidone (*n* = 41) or haloperidol (*n* = 37). Patients all had a 2-week washout period from previous antipsychotics before enrolment in the study. They found a reduction in the PANSS positive sub-score was negatively correlated with IL-2 at baseline (r = −0.36, *p* < 0.01) and IL-8 levels a baseline (r = −0.041, *p* = 0.016). No correlation was found for baseline IL-6. Based on a rating of responders (a minimum of 20% decrease in PANSS total score) and non-responders, only low baseline IL-8 remained significant (*p* = 0.036). In the same study, Zhang et al., 2009 [91], found a significant positive correlation between baseline superoxide dismutase (SOD) and the PANSS total score at the end of treatment (r = 0.27, *p* < 0.05), which shows the potential relationship between baseline oxidative stress markers and treatment effects.

##### Antipsychotics Not Specified

In an observational study, Chu et al., 2018 [92], investigated the effect of 8 weeks of antipsychotics (9 first-generation antipsychotic drugs and 21 second-generation anti-psychotic drugs) in drug-naïve (>2 weeks) schizophrenia patients (*n* = 30). They found correlations with baseline interleukin-1 receptor antagonist (IL-1ra) (r = −0.393, *p* = 0.032) and IL-6 (r = −0.407, *p* = 0.025) with changes in the Brief Psychiatric Rating Scale, Expanded (BPRS-E) manic scores, and insulin-like growth factor binding protein 3 (IGFBP3) (r = −0.446, *p* = 0.014) with changes in the Brief Psychiatric Rating Scale, Expanded (BPRS-E) anxiety scores. No significant correlation was found for the other sub-scores of BPRS (negative and positive) and BPRS total IL-1ra, IL-6, IGFBP3, and IFN-γ. The trial report measurements of additional immune biomarkers, i.e., IL-1β, IL-2, IL-8, IL-10, insulin-like growth factor binding protein 1 (IGFBP1), insulin-like growth factor binding protein 2 (IGFB2), BDNF, glial cell-derived neurotrophic factor (GDNF), nerve growth factor beta (Beta-NGF), and neural cell adhesion molecule (NCAM-1/CD56), but do not specify whether these measures were analysed for the correlation between baseline levels and treatment effect.

Mondelli et al., 2015 [93], investigated 68 first-episode psychosis patients in an observational study at baseline and at the end of 12 weeks of antipsychotic treatment. They assessed the following immune biomarkers: cortisol awakening response (CAR) IL-1β, IL-2, IL-4, IL-6, IL-8, IL-10, TNF-α, and IFN-γ. Only 39 patients were assessed at follow-up, and of them, 24 repeated the CAR (*n* = 12 responders and *n* = 12 non-responders) and 33 repeated the measurements of cytokines (16 responders and 17 non-responders). They used the Personal and Psychiatric History Schedule to classify responders and non-responders. A secondary analysis found that non-responders showed significantly lower CAR and had higher IL-6 and IFN-γ levels at baseline compared to responders. The remaining variables, IL-1β, IL-2, IL-4, IL-8, IL-10, and TNF-α, did not show differences between responders and non-responders at baseline. 

**Table 2:** Overview of studies on baseline immunological biomarkers and correlations with treatment response to antipsychotics included in this review.

Author	Country	Disease	Treatment	Treatment Length	N Analysed	Baseline Marker	Conclusion
Ma 2021 [81]	China	FES < 5 years	Olanzapine was added to the therapeutic dose (10–20 mg/day)	4 weeks	82	IL-1β, IL-6, IL-17, TGF-β_1_, hs-CRP	They found that responders had lower baseline IL-1β and IL-17, while no correlation was found for IL-6, hs-CRP, or TGF-β1.
Hatziagelaki 2019 [82]	Greece	Drug-naïve FES	Olanzapine was added to the therapeutic dose (15–20 mg/day)	8 weeks	14	IL-6 and IL-27, BDNF, IL-2, IL-17F, IL-17A, IL-22, IL-1β, IL-21, IL-23, IL-4, IFN-γ, TGF-β1, TGF-β2, and TGF-β3	IL-6 and IL-27 levels positively correlated to change in PANSS negative, while this association was not found for BDNF, IL-2, IL-17F, IL-17A, IL-22, IL-1β, IL-21, IL-23, IL-4, IFN-γ, TGF-β1, TGF-β2, and TGF-β3.
Lin 2017 [83]	China	SCZ	Olanzapine	4 weeks	38	IL-1β, TNF-α and MCP-1	No significant difference was found.
Lin 2017 [83]	China	SCZ	Risperidone	4 weeks	26	IL-1β, TNF-α, and MCP-1	Negative correlation between pre-treatment MCP-1 levels and PANNS-G reduction, while the correlation was not significant for total PANSS.
Susai 2023 [84]	Part of the OPTiMiSE study in 15 countries	FEP, SCZ, SCFD, or SCAD < 2 years	Amisulpride 200–800 mg/day, target dose 400 mg/day	4 weeks	294	Proteomics with multiple proteins	Complement C1R, C1S, C2, C5, C6, C7, C8A, C8B, CFI, FCN3, and MASP1 was associated with total PANSS.
Martinuzzi 2019 [85]	Part of the OPTiMiSE study in 14 countries	FEP, SCZ, SCFD, or SCAD < 2 years	Amisulpride 200–800 mg/day, target dose 400 mg/day	4 weeks	325	IL-1α, IL-1β, IL-2, IL-4, IL-5, IL-6, IL-7, IL-8, IL-10, IL-12p40, IL- 12p70, IL-13, IL-15, IL-16 IL- 17, IL-18, IL-21, IL-23, IL-27, IFN-γ, CCL2, CCL3, CCL4, CCL11, CCL13, CCL17, CCL19, CCL20, CCL22, CCL26, CCL27, CX3CL-1, CXCL10, CXCL11, CXCL12, TNF-α, TNF-β, GM-CSF, VEGF, CRP, SAA, sICAM-1, and sVCAM-1	In a subgroup of patients, correlation between low IL-15 at baseline and treatment response, as well as high CXCL12 at baseline and treatment response.
Crespo-Facorro 2008 [86]	Spain	Drug-naïve FEP	Risperidone 3–6 mg/day or olanzapine 5–20 mg/day or haloperidol 3–9 mg/day	6 weeks	56	IL-12	No significant associations between IL-12 production and changes in psychopathology.
Pae 2006 [87]	South Korea	Schizophrenia and drug-naïve at least 2 months	Risperidone or olanzapine or quetiapine	8 weeks	35	IL-2, IL-13, IL-6, IL-10 and TNF-α	No significant differences for baseline IL-2, IL-13, IL-6, IL-10, and TNF-α in responders vs. non-responders. Baseline IL-6 was correlated with a change in the PANSS-G score (r = 0.449, *p* = 0.044). No other correlation was found between changes in PANSS and baseline cytokine levels.
Zhang 2023 [88]	China	Schizophrenia	Risperidone or olanzapine or quetiapine or aripiprazole or ziprasidone or perphenazine or haloperidol	6 weeks	2598	Total WBC	Responders based on PANSS scores all had lower total WBC counts at baseline.
Zhang 2004, 2005, 2009 [89,90,91]	China	Schizophrenia	Risperidone 6 mg/day or haloperidol 20 mg/day	12 weeks	78	IL-2, IL-6, IL-8, and superoxide dismutase (SOD)	Negative correlation with serum IL-2 and IL-8 at baseline, no correlation was found for IL-6. Based on a rating of responder and non-responder low baseline IL-8 remained significant. SOD levels at baseline showed a significant positive correlation with PANSS total score.
Chu 2018 [92]	Taiwan	Schizophrenia and drug-naïve at least 2 weeks	Not specified	8 weeks	30	IL-1ra, IL-6, IGFBP3, and IFN-γ.	Negative correlations with baseline IL-1ra, IL-6, and IGFBP3 with changes in the BPRS-E manic and anxiety scores.
Mondelli 2015 [93]	UK	FEP	Not specified	12 weeks	39	Cortisol awakening response (CAR) IL-1β, IL-2, IL-4, IL-6, IL-8, IL-10, TNF-α, IFN-γ.	Non-responders at baseline had significantly lower CAR, and higher IL6 and IFN-γ levels when compared with responders

SCZ = schizophrenia; SCFD = schizophreniform disorder; SCAD = schizoaffective disorder; psychosis NOS = psychosis not otherwise specified; FEP = first-episode psychosis; FES = first-episode schizophrenia.

## 4. Discussion

To our knowledge, this is the first review investigating baseline immunological biomarkers as a predictor of the treatment effects of both anti-inflammatory add-on treatment to anti-psychotic treatment and the conventional antipsychotic treatment of psychotic disorders. Several inflammatory pathways have been implicated in psychotic disorders and the leading hypothesis is that anti-inflammatory add-on to antipsychotics will be efficient in only a subgroup of patients, which needs to be identified. The biomarkers identified in this review could represent predictors of treatment response aimed at personalizing current treatments by utilizing biomarkers for the stratification of patients. 

### 4.1. Immunological Biomarkers and the Prediction of Treatment Response to Anti-Inflammatory Add-on to Antipsychotics

Despite there being a substantial number of studies on anti-inflammatory add-on treatment to antipsychotics, only nine studies were identified that investigated correlations with baseline inflammatory biomarkers and treatment effects with seven different anti-inflammatory add-on treatments. The following baseline immunological markers showed significant associations with treatment response to add-on anti-inflammatory treatment in at least one of the studies’ exploratory analyses: CRP > 2 mg/L, IL-1β, IL-6, sTNF-R1, T_H_1/T_H_2 cytokine ratio (i.e., IFN-γ/IL-4), and GSH peroxidase activity. No significant difference were found for CRP > 1 mg/L, CRP >3.9 mg/L, CRP > 4 mg/L, IL-1β, IL-4, IL-6, IL-8, IL-10, IL-12, IL-17a, TNF-α and IFN-γ, CD4+, CD8+, CD19+, and sIL-2R [48,60,63,64,71,73,74,80]. Several previous meta-analyses and systematic reviews on anti-inflammatory add-on drugs to antipsychotics exist, but none have investigated baseline immunological biomarkers and the prediction of treatment response [39,40,41,42,43,44,45,46,47,49,50,52,53,54,55,56,94]. 

### 4.2. Immunological Biomarkers and the Prediction of Response to Treatment with Antipsychotics

The findings vary in terms of which antipsychotic is investigated and do not uniformly point in one direction for significant results. 

The studies showed significant findings in the prediction of treatment response to anti-psychotics for baseline WBC, IL-1ra, IL-1β, IL-2, IL-6, IL-8, IL-15, IL-17, IL-27, IFN-γ MCP-1, complement C1R, C1S, C2, C5, C6, C7, C8A, C8B, CFI, FCN3, MASP1, IGFBP3, CAR, SOD, and CXCL12 [81,82,83,84,85,88,89,90,91,92,93]. No significant differences were found for hs-CRP, CRP, IL-1α, IL-1β, IL-2, IL-4, IL-5, IL-6, IL-7, IL-8, IL-10, IL-12, IL-12p40, IL- 12p70, IL-13, IL-16, IL- 17,IL-17F, IL-17A, IL-18, IL-21,IL-22, IL-23, IL-27, IFN-γ, TNF-α, TNF-β, TGF-β1, TGF-β2, TGF-β3, MCP-1, BDNF, IGFB1, IGFB2, Beta-NGF, NMAC-1/CD56, CCL2, CCL3, CCL4, CCL11, CCL13, CCL17, CCL19, CCL20, CCL22, CCL26, CCL27, CX3CL-1, CXCL10, CXCL11, CXCL12, GM-CSF, VEGF, SAA, sICAM-1, sVCAM-1, and several complement proteins investigated in proteomics analyses [81,82,83,84,85,86,87,93]. It is worth noticing that many studies have investigated antipsychotics as a drug group and baseline markers as predictors of treatment response in the group, but the oversight of this paper indicates a need for individual analyses based on specific antipsychotic drugs. The most promising finding was in the clearly largest study of 2598 individuals, which found that responders to anti-psychotics had lower total WBCs at baseline.

### 4.3. Limitations and Strengths of Prior Studies Investigating Immunological Biomarkers as Predictors of Treatment Response to Anti-Inflammatory Add-on Treatment and Antipsychotics

The studies included in this review are overall small and underpowered for stratified analyses on immunological biomarkers at baseline for the prediction of treatment response, and, in addition, the investigated inflammatory biomarkers differ between the studies, making the current evidence base limited. Of the nine studies on anti-inflammatory add-on treatment included in this review, only one study (Weiser et al.) was powered sufficiently according to the author’s power analyses with the intention to compare treatment effects based on baseline inflammatory markers; in this case, CRP [48]. 

The study on prednisolone did not reach the estimated 88 patients to have at least 80% power to detect an effect size of 0.61. A study on aspirin by Laan et al. [60] was likewise underpowered, with only 70 patients, although their power analyses showed a need for 80 patients. Furthermore, the placebo group had a greater proportion of male patients, a shorter duration of illness, and a higher proportion of those treated with clozapine, which can influence results. In the study of NAC, the study population was new-onset psychosis within a year, but later on, diagnoses showed that three participants in the placebo group suffered from major depressive disorders as well as one in the intervention group [73]. In the study of tocilizumab, the authors questioned whether IL-6 is more of a state marker, and that the negative results of the trial might be due to only clinically stable patients being included in the trial since previous meta-analyses have shown IL-6 to be a state-dependent marker [14]. In the study of minocycline, fewer patients than planned were kept in the study; however, the study is the one with the longest (12 months) follow-up time, and when assessing the 2-month and 3-month follow-ups, a larger proportion was retained, which makes the statical analysis more valid [64]. The study on celecoxib lacks demographical data beyond sex and has a short treatment period of only 5 weeks [63]. In the Pravastatin study, there was a larger proportion of patients receiving two antipsychotics in the intervention group vs. the placebo group (12 vs. 4, *p* = 0.04), which could be due to differences in the severity of illness, which can affect the variable of immunological markers [16,18,30]. 

Demographical differences among the diagnoses of study participants in the studies included, and treatment status (first episode, stable, or relapse), result in low comparability between the included studies. If the hypothesis is that neuroinflammation causes the psychosis, then in stable patients, we might be at a point past the primary inflammation, and an anti-inflammatory add-on might therefore only be beneficial in first-time psychosis, which is supported by previous meta-analyses showing a state-dependent increase, especially in first-episode psychosis. 

The continued use of antipsychotics during and before the studies on anti-inflammatory add-ons can cause a ceiling effect in studies including clinically stable patients, thus smaller effect differences can be difficult to show in these small studies. Dose-dependent studies are also lacking in the field of anti-inflammatory add-on drugs, where higher or lower doses might be needed [63]. Previous studies and the studies included in this review on anti-psychotics have shown various effects on immunological biomarkers; therefore, in a group with various uses of antipsychotics, this might influence the results [57,95,96]. Only one study of anti-inflammatory add-on treatment had a washout period from previous antipsychotics to eliminate any influence on the results in the group of anti-inflammatory add-on trials [63].

Further biases to the results are potential poor treatment adherence, interrater differences, the genetic aspects of the participants, a lack of adjustment for confounders such as smoking status [97] and BMI [98,99], sex, and age, which might be associated with differentiated expressions of biomarkers. Moreover, studies investigating long-term outcomes in anti-inflammatory add-ons to antipsychotics are lacking. The conclusions from the findings among the studies included in this review are unclear concerning which biomarker values are considered to be high or low, and in the statistical analyses, the results on the correlation of baseline inflammatory biomarkers and treatment effect are primarily found in a secondary analysis and in some of the studies are only mentioned in the discussion part of the paper. Moreover, some of the studies are more than 20 years old and methods to detect cytokines have become better over time.

In this study, we have not explored safety measures or adverse events in the drugs investigated, but a future implication of anti-inflammatory add-on drugs should always consider adverse events.

## 5. Perspectives

Potential future applications could be the utilization of baseline immunological biomarkers for the prediction of the choice of treatment and the prediction of treatment efficacy for first-episode psychosis—or a prediction of which treatment would lead to higher treatment effects on specific psychiatric symptoms such as the positive symptoms, negative symptoms, or total psychopathology. Between 6 and 19% of patients with first-episode psychosis are initially diagnosed with acute and transient psychotic disorders and approximately 44% among this group will have a diagnostic shift in this group within 2 years, mainly towards schizophrenia [100]. There are differences in pro-inflammatory cytokines in patients with acute and transient psychotic disorders in remission (as well as in the acute phase) compared to patients with schizophrenia [101]. Although the findings are from a small exploratory study, these findings could possibly lead to the early identification of patients who will stay stable from long-lasting psychotic disorders and could thus impact treatment decisions. Possible preventive measurements in the field of clinical high-risk state for psychosis could be helped with the same markers for the stratification of the predicted course of illness [102]. Studies taking multiple baseline markers as predictors and using machine learning-estimated responses to treatment could be an approach leading to new, innovative personalized treatments. Large-scale RCTs approaching precision medicine are needed and studies investigating this are ongoing [103]. 

Similar approaches could be extended to other categories of mental disorders, such as mood disorders. Previous studies examining the use of anti-inflammatory add-on treatments in mood disorders have shown higher efficacy in patients displaying signs of inflammation at baseline. Here, one study on infliximab therapy in depressed patients found that treatment response based on the Hamilton Depression Rating Scale 17 (HAM-D-17) only showed improvement in patients with hs-CRP > 5 mg/L [104]. In another study, responders (at least a 25% reduction on HAM-D-17) to minocycline on depression was observed specifically within the subgroup of patients with baseline CRP > 3 mg/L and baseline IL-6 predicted treatment response [105], and a different study on minocycline in bipolar depression found that patients with elevated baseline IL-6 levels responded better to minocycline [106]. However, all these findings were exploratory and none of these studies had the stratification of patients by immunological markers as a primary outcome.

## 6. Conclusions

In this review, we present an overview of the published literature on immunological biomarkers as predictors of treatment response to antipsychotics and anti-inflammatory add-on treatment for psychotic disorders. The review identified several baseline immunological biomarkers as relevant for the prediction of treatment response; however, studies investigating this as a primary outcome are lacking and current studies possess several limitations. 

To advance precision medicine approaches of utilizing biomarkers as predictors of treatment effect, individual participant data meta-analyses [107] would be a first step, but larger comprehensive RCTs powered to investigate baseline biomarkers for stratification as a primary outcome regarding anti-inflammatory add-on treatment but also antipsychotics are vastly lacking. Identifying objective markers as immunological biomarkers predictive of treatment response to a given type of treatment for psychotic disorders holds the potential to enhance and individualize treatment approaches in psychotic disorders, hopefully leading to overall better treatment response. 
